# Optical Coherence Tomography Artifacts Are Associated With Adaptive Optics Scanning Light Ophthalmoscopy Success in Achromatopsia

**DOI:** 10.1167/tvst.10.1.11

**Published:** 2021-01-07

**Authors:** Katie M. Litts, Erica N. Woertz, Michalis Georgiou, Emily J. Patterson, Byron L. Lam, Gerald A. Fishman, Mark E. Pennesi, Christine N. Kay, William W. Hauswirth, Michel Michaelides, Joseph Carroll

**Affiliations:** 1 Department of Ophthalmology & Visual Sciences, Medical College of Wisconsin, Milwaukee, WI, USA; 2 Department of Cell Biology, Neurobiology and Anatomy, Medical College of Wisconsin, Milwaukee, WI, USA; 3 UCL Institute of Ophthalmology, University College London, London, UK; 4 Moorfields Eye Hospital NHS Foundation Trust, London, UK; 5 Bascom Palmer Eye Institute, University of Miami, Miami, FL, USA; 6 Pangere Center for Inherited Retinal Diseases, The Chicago Lighthouse, Chicago, IL, USA; 7 Casey Eye Institute, Oregon Health & Science University, Portland, OR, USA; 8 Vitreoretinal Associates, Gainesville, FL, USA; 9 Department of Ophthalmology, University of Florida, Gainesville, FL, USA

**Keywords:** achromatopsia, optical coherence tomography, adaptive optics, inherited retinal disease

## Abstract

**Purpose:**

To determine whether artifacts in optical coherence tomography (OCT) images are associated with the success or failure of adaptive optics scanning light ophthalmoscopy (AOSLO) imaging in subjects with achromatopsia (ACHM).

**Methods:**

Previously acquired OCT and non-confocal, split-detector AOSLO images from one eye of 66 subjects with genetically confirmed achromatopsia (15 *CNGA3* and 51 *CNGB3*) were reviewed along with best-corrected visual acuity (BCVA) and axial length. OCT artifacts in interpolated vertical volumes from CIRRUS macular cubes were divided into four categories: (1) none or minimal, (2) clear and low frequency, (3) low amplitude and high frequency, and (4) high amplitude and high frequency. Each vertical volume was assessed once by two observers. AOSLO success was defined as sufficient image quality in split-detector images at the fovea to assess cone quantity.

**Results:**

There was excellent agreement between the two observers for assessing OCT artifact severity category (weighted kappa = 0.88). Overall, AOSLO success was 47%. For subjects with OCT artifact severity category 1, AOSLO success was 65%; for category 2, 47%; for category 3, 11%; and for category 4, 0%. There was a significant association between OCT artifact severity category and AOSLO success (*P* = 0.0002). Neither BCVA nor axial length was associated with AOSLO success (*P* = 0.07 and *P* = 0.75, respectively).

**Conclusions:**

Artifacts in OCT volumes are associated with AOSLO success in ACHM. Subjects with less severe OCT artifacts are more likely to be good candidates for AOSLO imaging, whereas AOSLO was successful in only 7% of subjects with category 3 or 4 OCT artifacts. These results may be useful in guiding patient selection for AOSLO imaging.

**Translational Relevance:**

Using OCT to prescreen patients could be a valuable tool for clinical trials that utilize AOSLO to reduce costs and decrease patient testing burden.

## Introduction

Clinical trials and natural history studies for inherited retinal diseases such as achromatopsia (ACHM) are increasingly utilizing adaptive optics scanning light ophthalmoscopy (AOSLO) to assess cone structure.^1^ The single-cell resolution afforded by AOSLO imaging provides information that is relevant for these gene therapy studies, as cones represent the cellular target for such therapeutic efforts. Additionally, AOSLO imaging could play a role in monitoring the safety and efficacy of other therapeutic interventions aimed at restoring cone function and/or slowing cone degeneration. As such, there is interest in expanding the clinical accessibility and utilization of AOSLO; however, recent studies have shown that usable AOSLO images for which cone density could be generated were obtained in just over half of subjects with ACHM.^2^^–^^4^ The relatively low success of AOSLO in this patient population results in a waste of time and effort on the part of both the patient and the researcher. Additionally, many patients must travel long distances to complete AOSLO imaging, which imposes a financial burden for study teams. The ability to determine which patients will provide usable AOSLO images could lead to considerable savings for funding agencies, pharmaceutical companies, researchers, and patients alike.

A major limiting factor in obtaining high-quality AOSLO images is the presence of nystagmus.^2^^,^^3^ Thus, one approach could be to pre-screen the severity of nystagmus prior to referral for AOSLO imaging. Although there are many ways to visualize and quantify nystagmus, including eye tracking and electrooculography,^5^^,^^6^ additional clinical assessments would be somewhat counterproductive to the goal of reducing waste and increasing efficiency. In contrast to these techniques, optical coherence tomography (OCT) is already widely used to visualize the retina in a number of inherited retinal diseases, including ACHM.^7^^–^^10^ Clinical OCT images contain numerous artifacts, or vertical discontinuities; although many relate to the segmentation of the image,^11^ others arise from involuntary eye movements during image acquisition.^12^ There has been significant effort aimed at reducing these motion artifacts through both software and hardware strategies, as they are generally viewed as an impediment for the clinical utilization of OCT imagery.^12^^–^^14^

Although these motion artifacts can make clinical interpretations of OCT images challenging, these artifacts may contain potentially useful information about underlying eye movements. Therefore, we hypothesized that grading of OCT artifacts could be used to assess the relative severity of eye movements and thus be associated with whether AOSLO imaging would be successful in a given patient. As OCT can be acquired in nearly every patient (even those with nystagmus), this approach could serve as a valuable tool for clinical trials that utilize AOSLO to reduce costs and decrease patient testing burden.

## Methods

### Subjects

This research followed the tenets of the Declaration of Helsinki and was approved by the Institutional Review Board at the Medical College of Wisconsin (PRO00030741). Written informed consent was obtained from all participants and their information stored in a database (Lattice Version 1.0; Translational Imaging Innovations, Inc., Hickory, NC). Images from 66 patients (mean age, 22.5 years; range, 7–57 years; 30 females) with genetically confirmed *CNGA3*- or *CNGB3*-associated ACHM (15 and 51 subjects, respectively) were used for this study (Supplementary Table S1). The right eye of each subject was included, unless only the left eye was imaged or was previously reported. There were 48 subjects who were recruited as part of other studies and have appeared in previous publications (Supplementary Table S2).^2^^–^^4^^,^^10^^,^^15^^–^^20^ Best-corrected visual acuity (BCVA) was collected for the eye included in this study using either the Early Treatment Diabetic Retinopathy Study chart or the electronic visual acuity protocol. Axial length measurements from a Zeiss IOLMaster (Carl Zeiss Meditec, Dublin, CA) were also extracted for use in this analysis.

### OCT Imaging and Artifacts

**Figure 1.**
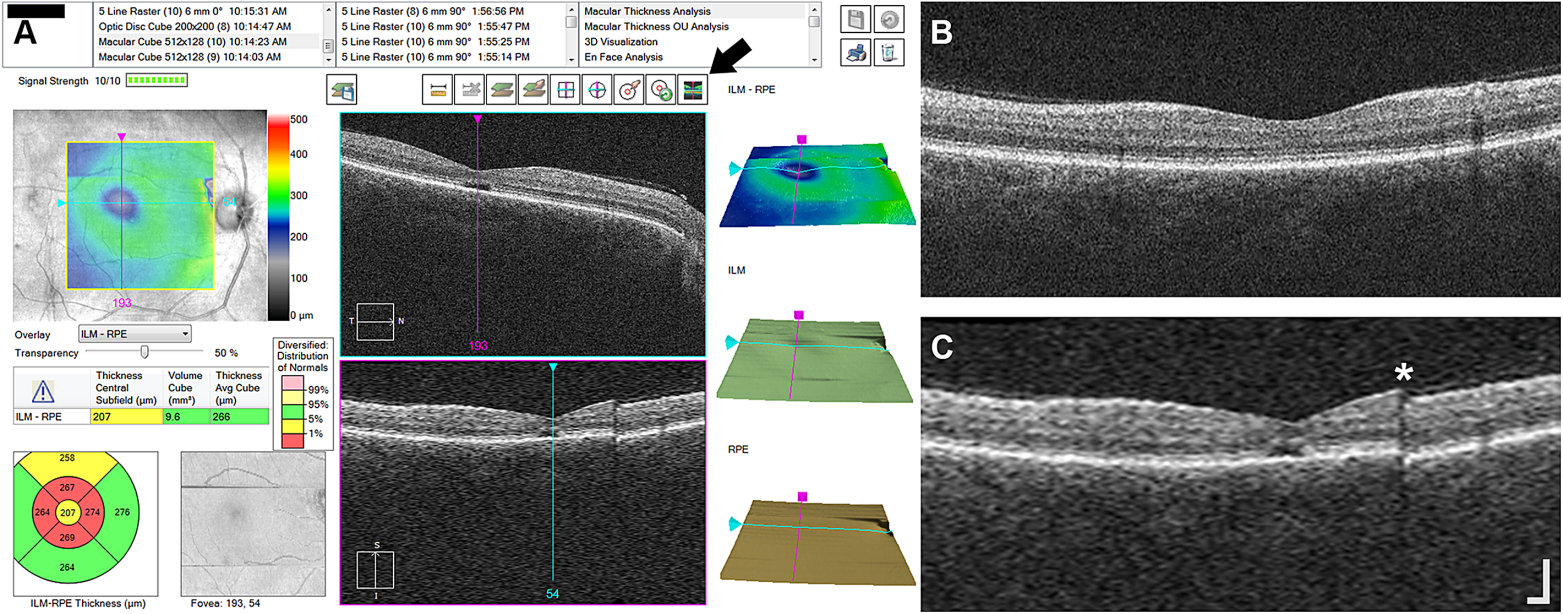
OCT artifacts in interpolated vertical scans on CIRRUS. (**A**) Screenshot of the “Macular Thickness Analysis” interface on a CIRRUS HD-OCT device for subject JC_11062. Horizontal B-scans from the macular cube (512 A-scans, 128 B-scans) are displayed in the top middle panel. Interpolated vertical scans from the vertical volume are displayed in the bottom middle panel. The vertical volume is interpolated data between the 128 B-scans in the macular cube volume. Pressing the button noted by the arrow will display the vertical high-resolution B-scan in the bottom middle panel instead of the interpolated vertical scan. (**B**) Vertical high-resolution B-scan from JC_11062. (**C**) Interpolated vertical scan (“slow scan”) showing a clear artifact (asterisk) that was not captured in the fast high-resolution scan in B. This vertical volume was assessed as OCT severity category 2. *Scale bar*: 200 µm.

**Figure 2.**
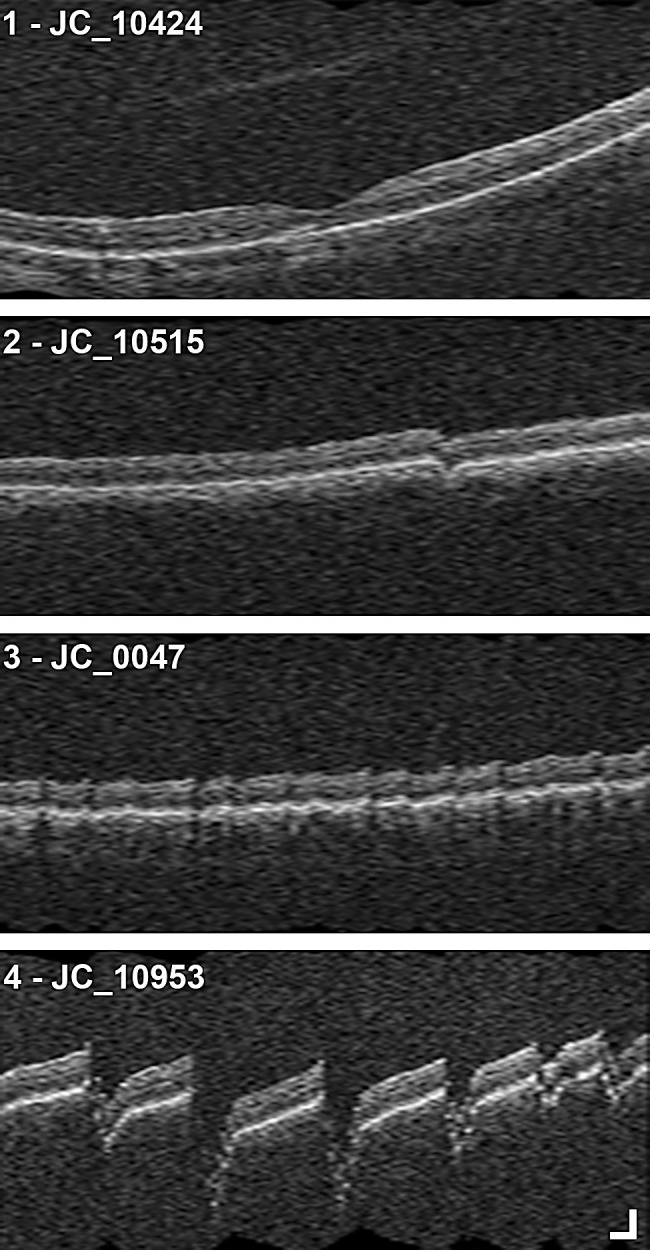
OCT artifact severity categories. Example interpolated vertical scans from CIRRUS macular cubes (512 A-scans, 128 B-scans) from four subjects showing artifacts for each of the four OCT artifact severity categories: (1) artifacts are not present or minimal, (2) artifacts are clear and low frequency, (3) artifacts are low amplitude and high frequency, and (4) artifacts are high amplitude and high frequency. *Scale bar*: 200 µm.

Previously acquired OCT images from one eye of all subjects with ACHM were used for analysis. Prior to imaging, one eye from each subject with ACHM was dilated using either a single drop of cyclomydril (cyclopentolate hydrochloride, 0.2%; phenylephrine hydrochloride, 1%) or a combination of tropicamide (1%) and phenylephrine hydrochloride (2.5%) for cycloplegia and pupillary dilation. A macular cube (512 A-scans, 128 B-scans; nominal scan size, 6 mm × 6 mm) was acquired using the CIRRUS HD-OCT (Carl Zeiss Meditec). On the CIRRUS device, vertical volumes were reviewed on the “Macular Thickness Analysis” interface as shown in Figure 1. As the slow scan acquisition is along the superior–inferior meridian, the vertical B-scans in the macular cube are not true B-scans but instead are constructed by interpolating data between the 128 B-scans in the volume. For each volume, the interpolated vertical B-scans were reviewed for OCT artifacts, visible discontinuities in the vertical scan as a result of atypical axial displacement between adjacent horizontal B-scans. The OCT artifacts were divided into one of four severity categories: (1) none or minimal, (2) clear and low frequency, (3) low amplitude and high frequency, and (4) high amplitude and high frequency (Fig. 2). Each interpolated vertical volume was assessed once by two observers (KML and ENW). A third observer (JC) assessed any discrepancies and reviewed any additional volumes when needed to reach a consensus, which was used for subsequent analyses.

### AOSLO Imaging and Success

Previously acquired non-confocal split-detector AOSLO images and montages were used for this study, as described elsewhere.^2^^,^^4^ AOSLO success was defined as a montage of split-detector images at the fovea having sufficient quality to assess cone quantity. AOSLO raw videos (for examples of AOSLO success and failure, see Supplementary Movie S1 and Supplementary Movie S2, respectively) that were unable to be processed, processed images that were unable to be montaged, or montages containing images with poor quality where cones could not be identified reliably were defined as AOSLO failure. For previously unpublished AOSLO data (20 subjects; Supplementary Table S2), montages were unable to be generated due to poor image quality in nine subjects. The remaining 11 subjects with montages were graded by two observers (KML and MG), and a third observer (EJP) resolved ambiguities (five subjects). AOSLO assessment was completed prior to OCT evaluation and observer KML was masked to the outcome for AOSLO when assessing OCT artifacts. Subjects were grouped by AOSLO success/failure for comparison of OCT artifact severity categories, as described above.

### Statistical Analysis

To assess the reliability of classifying subjects into OCT artifact severity categories, Cohen's weighted kappa test^21^ was used (GraphPad Software, La Jolla, CA). The χ^2^ test for trend (GraphPad Prism 8) was used to detect a trend between OCT artifact severity categories and AOSLO success.

## Results

Using our OCT artifact severity categories (Fig. 2), there was excellent agreement between the assessments of two observers (weighted kappa = 0.88, 95% confidence interval, 0.80–0.97). Of the 66 subjects evaluated by the two observers, there were six discrepancies in the assessments. Evaluation of these discrepancies by a third observer determined three subjects with category 1 (two observers assessed as categories 1 and 2 for two subjects and categories 2 and 3 for one subject), two subjects with category 2 (two observers assessed as categories 2 and 3 for one subject and categories 2 and 4 for one subject), and one subject with category 4 (two observers assessed as categories 4 and 3). The final OCT artifact severity category assessment resulted in 34 subjects with category 1 (minimal or no artifacts), 17 subjects with category 2 (clear and low-frequency artifacts), nine subjects with category 3 (low-amplitude and high-frequency artifacts), and six subjects with category 4 (high-amplitude and high-frequency artifacts).

**Figure 3.**
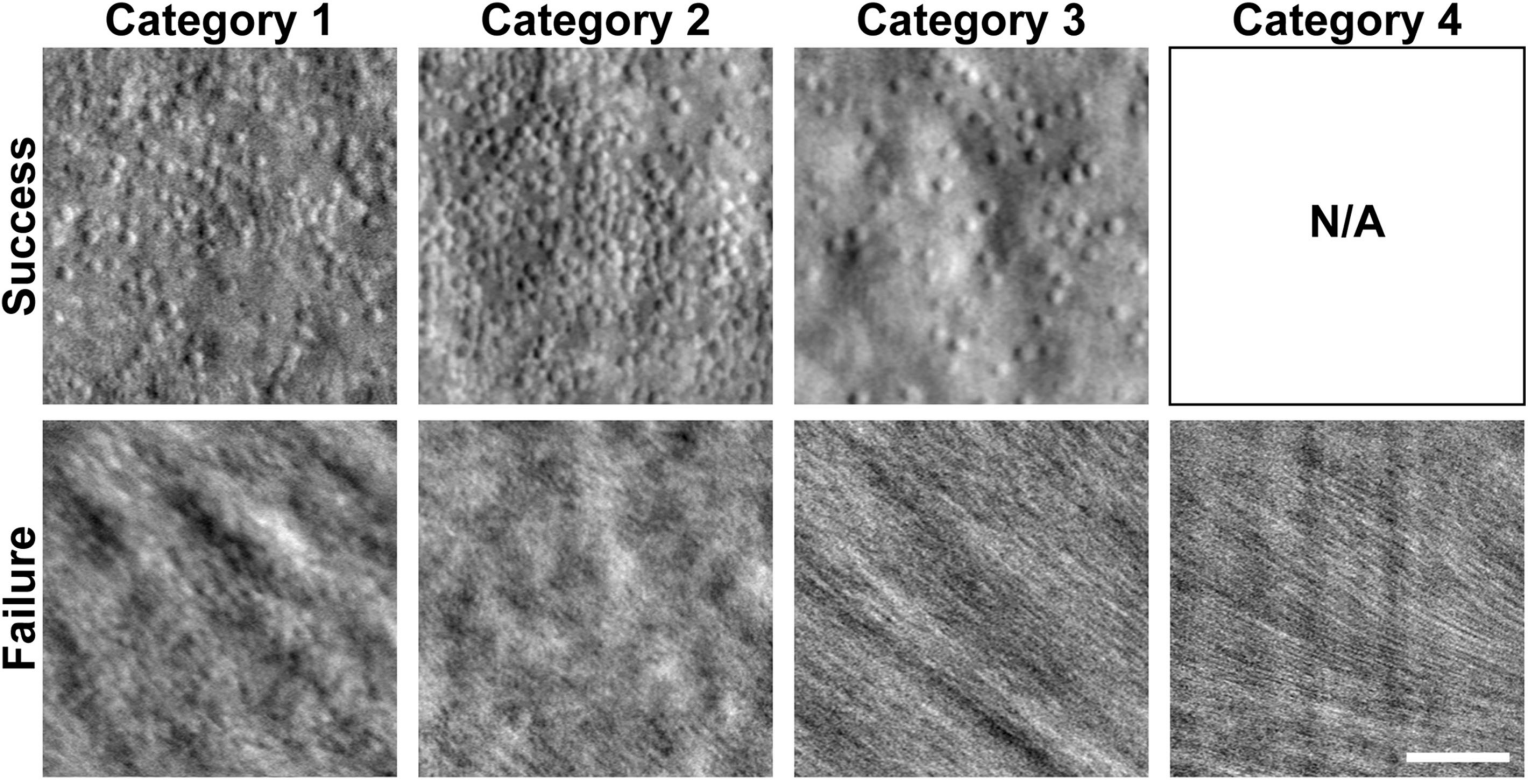
Example split-detector AOSLO images for each OCT artifact severity category. AOSLO images assessed as success (*top row*) and failure (*bottom row*) are shown for each OCT artifact severity category (*columns*). Successful AOSLO images were extracted from the location of peak cone density, whereas the failed AOSLO images were the best registered TIFF images processed from videos acquired at foveal fixation. There were no subjects with successful AOSLO images for category 4. N/A, not applicable. *Scale bar*: 50 µm.

**Figure 4.**
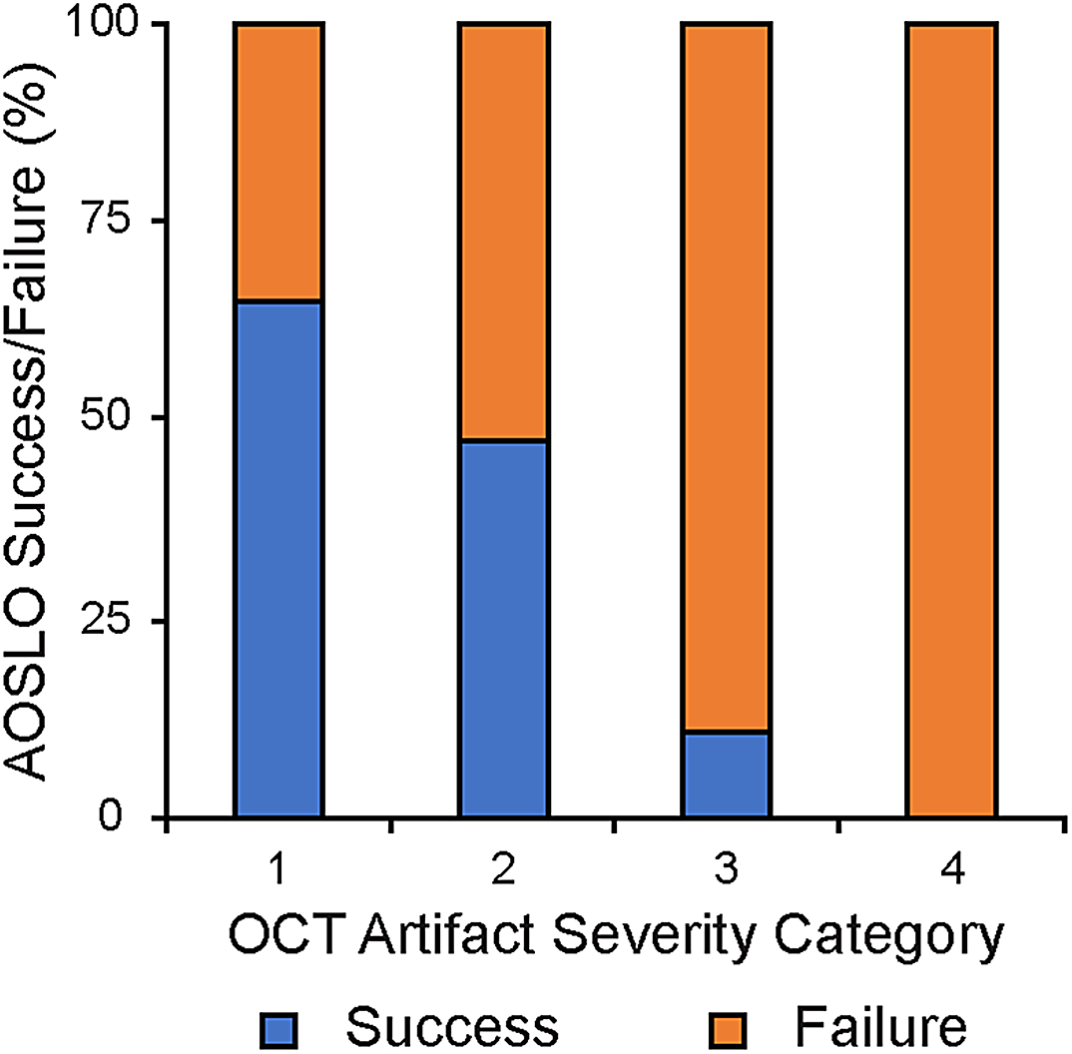
OCT artifact severity category is associated with AOSLO success. Shown is the percent AOSLO success (*blue*) and failure (*orange*) for subjects within each OCT artifact severity category. There was a significant association between OCT artifact severity category and AOSLO success (*P* = 0.0002, χ^2^ test).

AOSLO success was assessed for the 66 subjects in this study (as described in the Methods). Overall, AOSLO was successful in 47% (31/66) of subjects. For subjects with OCT artifact severity category 1, AOSLO success was 65% (22/34 subjects); for category 2, 47% (8/17 subjects); for category 3, 11% (1/9 subjects); and for category 4, 0% (0/6 subjects). Example AOSLO images for each category are shown in Figure 3. There was a significant association between OCT artifact severity category and AOSLO success (*P* = 0.0002, χ^2^) (Fig. 4).

BCVA and axial length are other measures that are often collected clinically and may relate to the ability to obtain successful AOSLO images. In this study, BCVA was not significantly different between the AOSLO success and AOSLO failure groups: AOSLO success mean ± SD = 0.85 ± 0.16 logMAR; AOSLO failure mean ± SD = 0.88 ± 0.12 logMAR (*P* = 0.07, Mann–Whitney test). In addition, axial length was not significantly different between the AOSLO success and AOSLO failure groups: AOSLO success mean ± SD = 24.10 ± 1.99 mm; AOSLO failure mean ± SD = 23.95 ± 1.97 mm (*P* = 0.75, unpaired *t*-test).

## Discussion

Here, we demonstrate that OCT images contain information that may be used to determine the success of AOSLO imaging in subjects with ACHM. Our OCT artifact severity categories correlate with AOSLO success, as subjects with less severe categories (1 and 2) are likely to be better candidates for AOSLO imaging than subjects with high-severity categories (3 and 4). OCT artifact severity category 1 had higher AOSLO success than previously reported overall AOSLO success in similar cohorts that noted nystagmus being the limiting factor in acquiring analyzable AOSLO images.^2^^,^^3^ Although OCT artifact severity category 4 was always associated with AOSLO failure, our data show that OCT artifact severity category 1 was not always associated with AOSLO success. This may be due to other factors that could affect AOSLO image quality such as tear film and the optics of the eye but could also be due to the subjective nature of our OCT grading scheme. Factors such as BCVA and axial length were not associated with AOSLO success for the subjects included in this study. More direct and quantitative measures of eye (or retinal) motion, such as eye tracking or electrooculography,^5^^,^^6^ may be worth examining. Although these methods might correlate better with AOSLO success, it would come at the cost of increased patient testing burden. Regardless of the method used, nystagmus has been reported to improve with age in patients with ACHM.^18^^,^^22^ Thus, the likelihood that AOSLO success may change over time for a given patient has important implications for longitudinal clinical trials.

Among the strengths of our study are the use of standardized protocols for data acquisition including the same AOSLO system and the same OCT device. We investigated a large number of genetically confirmed patients with the two most common genotypes associated with ACHM. We used at least two observers, experienced in OCT analysis, for assessing artifacts and a third observer when needed to resolve discrepancies. In addition to its strengths, our study had several limitations. First, only subjects with *CNGA3*- and *CNGB3*-associated ACHM were included. The observed association between AOSLO success and the OCT artifact severity category may not be generalized to other ACHM genotypes. For example, other studies reported AOSLO success for *ATF6*-associated ACHM as 57% (4/7 subjects)^19^; for *PDE6C*-associated ACHM as 33% (2/6 subjects), with only one of the four AOSLO failures being related to nystagmus^23^; and for *GNAT2*-associated ACHM as 83% (5/6 subjects).^24^ Second, OCT artifact severity categories were developed using volumes from a CIRRUS device and may not translate to volumes acquired using eye-tracking or other devices. As OCT devices continue to evolve with increased scanning speed (2.4 seconds per volume for the CIRRUS device in this study) and larger imaging areas,^25^ it might not be possible to exploit OCT for the purpose used in this study, as such technological advances would likely decrease the amount of visible artifacts present as a result of unstable fixation or eye motion. Third, these findings may apply only to the Medical College of Wisconsin (MCW) AOSLO system,^16^^,^^26^ as there is extensive variability in AOSLO hardware and a lack of its convergence among research groups.^27^ Finally, there may be differences in a subject's fixation between OCT and AOSLO, which utilize different fixation targets, that may impact eye motion between devices.

In spite of these limitations, our study demonstrated that patients for whom AOSLO was unsuccessful can provide important insights into existing imaging technologies and how they might be applied or improved for patients with nystagmus. With the increased resolution and decreased field of view afforded by AOSLO compared to OCT, unstable fixation, eye motion, and nystagmus can cause significant distortions in single images acquired in an imaging sequence (at 16.6 frames per second for the MCW AOSLO) due to the scanning nature of the device. Taking advantage of this, AOSLO images themselves can be used to produce accurate motion traces (Kane T, et al. *IOVS*. 2019;60:ARVO E-Abstract 4605).^28^^,^^29^ Correlating this high-resolution information with other clinical measures could predict which clinical screening measure may be helpful in screening patients, thus directing hardware and/or software improvements for AOSLO. For example, it may be possible that systems that allow for larger fields of view or increased frame rate may increase the AOSLO success rate when it comes to higher frequency nystagmus. Other groups have developed systems with increased image acquisition to accommodate eye movements up to 100 Hz^30^ and the ability to correct eye movements in real time.^31^ Although such system upgrades could be costly upfront, quantitative data about those patients with failed AOSLO imaging could direct this effort and thus could increase the AOSLO success in patients with ACHM.

As OCT is acquired in nearly every patient with an inherited retinal disorder, our approach could be extended to other populations where the presence of nystagmus interferes with AOSLO image acquisition, such as albinism.^32^ However, the pattern of eye movements is likely different in these other conditions,^33^ so it would be necessary to develop screening methods specific to particular diseases. Beyond conditions associated with nystagmus in which AOSLO imaging is challenging (e.g., media opacities, dry eye, unstable fixation), it may be possible to apply a similar prescreening approach using information already available in OCT images. For example, media opacities have been shown to decrease reflectivity in OCT images,^34^ and the presence of cataracts has been shown to decrease OCT signal quality.^35^^,^^36^ There are robust methods to quantify OCT signal quality^37^ that would be worth exploring as complementary predictors of AOSLO imaging success. In addition, a prescreening method could also indicate the type of AO system that might lead to successful images, such as using a wavefront sensorless system in cases of cataracts.^38^ A comprehensive effort to develop effective prescreening strategies would serve to reduce the waste of valuable research resources, as well as increase the overall AOSLO data yield in trials. As AOSLO systems continue to improve, developing methods to prospectively identify the subset of patients in which imaging may fail will be important, given the limited availability of AOSLO systems.

## Supplementary Material

Supplement 1

Supplement 2

Supplement 3

Supplement 4

## Supplementary Material

**Supplementary Movie S1**. Example raw AOSLO video from a subject with analyzable images.

**Supplementary Movie S2**. Example raw AOSLO video from a subject with images that could not be processed due to severe nystagmus.
