# Supplementary figures and images for: Optical Coherence Tomography Artifacts Are Associated With Adaptive Optics Scanning Light Ophthalmoscopy Success in Achromatopsia
Source: Transl Vis Sci Technol. 2021 Jan 7;10(1):11. doi: 10.1167/tvst.10.1.11 (PMC7804582; doi:10.1167/tvst.10.1.11)

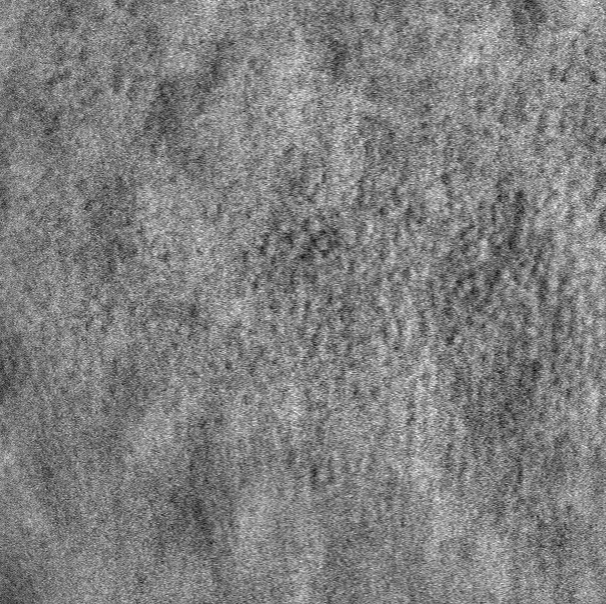

Supplement: Supplement 1 [file tvst-10-1-11_s001.gif]

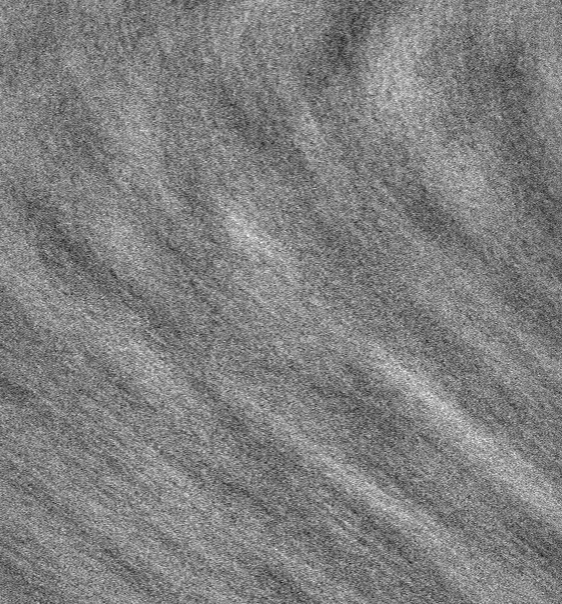

Supplement: Supplement 2 [file tvst-10-1-11_s002.gif]
