# Supplementary material for: Optical Coherence Tomography Artifacts Are Associated With Adaptive Optics Scanning Light Ophthalmoscopy Success in Achromatopsia
Source: Transl Vis Sci Technol. 2021 Jan 7;10(1):11. doi: 10.1167/tvst.10.1.11 (PMC7804582; doi:10.1167/tvst.10.1.11)
Supplement: Supplement 3 [file tvst-10-1-11_s003.pdf]

Supplementary Table S1. Subject demographics and data

Subject ID	Genotype	Gender	Age (years)	Eye	AOSLO Success	OCT Artifact Severity Category
JC_0047*	*CNGB3*	M	18	OD	Y	1
JC_10028	*CNGB3*	F	13	OD	Y	1
JC_10069	*CNGA3*	M	18	OD	Y	1
JC_10167	*CNGB3*	F	17	OD	Y	1
JC_10196	*CNGB3*	F	34	OD	Y	1
JC_10197	*CNGB3*	F	9	OD	Y	1
JC_10216	*CNGB3*	M	17	OD	Y	1
JC_10224	*CNGB3*	M	38	OD	Y	1
JC_10232	*CNGB3*	M	19	OD	Y	1
JC_10247	*CNGB3*	M	25	OD	Y	1
JC_10260	*CNGB3*	M	31	OD	Y	1
JC_10335	*CNGB3*	F	18	OD	Y	1
JC_10409	*CNGB3*	F	32	OD	Y	1
JC_10417	*CNGB3*	F	23	OS	Y	1
JC_10853	*CNGB3*	F	28	OD	Y	1
JC_10854	*CNGB3*	M	32	OD	Y	1
JC_11067	*CNGA3*	M	24	OD	Y	1
JC_11155	*CNGA3*	F	31	OD	Y	1
JC_11228	*CNGA3*	F	29	OD	Y	1
JC_1208	*CNGB3*	M	18	OD	Y	1
JC_11034	*CNGB3*	F	46	OD	Y	1
JC_11066	*CNGA3*	F	11	OD	Y	1
JC_10198	*CNGB3*	F	44	OD	N	1
JC_10213	*CNGB3*	M	12	OD	N	1
JC_10215*	*CNGB3*	M	14	OD	N	1
JC_10217	*CNGB3*	F	8	OD	N	1
JC_10249	*CNGB3*	M	16	OD	N	1
JC_10258	*CNGB3*	F	43	OD	N	1
JC_10334	*CNGB3*	M	22	OD	N	1
JC_10617	*CNGA3*	M	15	OD	N	1
JC_11296	*CNGA3*	M	12	OD	N	1
JC_11579	*CNGB3*	M	9	OD	N	1
JC_11623	*CNGA3*	M	15	OD	N	1
KS_11531	*CNGB3*	M	24	OD	N	1
JC_10151	*CNGB3*	F	11	OD	Y	2

JC_10191*	*CNGB3*	M	36	OD	Y	2
JC_10248	*CNGB3*	F	16	OD	Y	2
JC_10310	*CNGB3*	M	33	OD	Y	2
JC_10416	*CNGB3*	F	30	OS	Y	2
JC_10424	*CNGB3*	M	27	OD	Y	2
JC_11062	*CNGA3*	F	41	OD	Y	2
KS_10337	*CNGA3*	F	17	OD	Y	2
JC_10142	*CNGB3*	F	45	OD	N	2
JC_10195	*CNGB3*	M	9	OD	N	2
JC_10214	*CNGB3*	M	10	OD	N	2
JC_10300*	*CNGB3*	M	9	OD	N	2
JC_10968	*CNGB3*	M	43	OD	N	2
JC_10999	*CNGB3*	F	10	OD	N	2
JC_11060	*CNGA3*	F	57	OD	N	2
JC_11401	*CNGA3*	M	24	OD	N	2
JC_10024	*CNGB3*	M	28	OD	N	2
JC_10089*	*CNGB3*	F	40	OD	Y	3
JC_10025	*CNGB3*	M	19	OD	N	3
JC_10168	*CNGB3*	F	10	OD	N	3
JC_10250	*CNGB3*	M	18	OD	N	3
JC_10257*	*CNGB3*	M	14	OD	N	3
JC_10490	*CNGB3*	F	49	OD	N	3
JC_10551	*CNGA3*	M	10	OS	N	3
JC_11036	*CNGB3*	F	9	OD	N	3
KS_11453	*CNGA3*	M	18	OD	N	3
JC_10256	*CNGB3*	F	23	OD	N	4
JC_10029	*CNGB3*	F	12	OD	N	4
JC_10953	*CNGB3*	M	16	OD	N	4
JC_11000	*CNGB3*	M	18	OD	N	4
JC_11061*	*CNGB3*	F	7	OD	N	4
JC_11297	*CNGA3*	M	12	OD	N	4

*Subject included in Figure 3.
M=Male; F=Female
