# Supplementary material for: Optical Coherence Tomography Artifacts Are Associated With Adaptive Optics Scanning Light Ophthalmoscopy Success in Achromatopsia
Source: Transl Vis Sci Technol. 2021 Jan 7;10(1):11. doi: 10.1167/tvst.10.1.11 (PMC7804582; doi:10.1167/tvst.10.1.11)
Supplement: Supplement 4 [file tvst-10-1-11_s004.pdf]

Supplementary Table S2. Previous reporting of ACHM subjects

**Subject ID in Current Study**	Dubis et al (2014)[1]	Scoles et al (2014)[2]	Abozaid et al (2016)[3]	Langlo et al (2016)[4]	Langlo et al (2017)[5]	Georgiou et al (2019)[6]	Mastey et al (2019)[7]	Mastey et al (2019)[8]	Litts et al (2020)[9]	Georgiou et al (2020)[10]
JC_0047				PCI-007	PCI-007				same ID	
JC_10028		same ID		PCI-004	PCI-004					
JC_10069		same ID	same ID			same ID		same ID	same ID	same ID
JC_10167				BPE-003	BPE-003				same ID	
JC_10196				UFC-002	UFC-002				same ID	
JC_10197				UFC-004	UFC-004				same ID	
JC_10216				PCI-013	PCI-013				same ID	
JC_10224				PCI-021	PCI-021				same ID	
JC_10232				CEI-001	CEI-001			same ID	same ID	
JC_10247				UFC-001	UFC-001				same ID	
JC_10260				CEI-006	CEI-006					
JC_10335				PCI-025						
JC_10409				PCI-031	PCI-031					
JC_10417				BPE-019	BPE-019					
JC_10853				PCI-032					same ID	
JC_10854				PCI-033					same ID	
JC_11067										
JC_11155										
JC_11228										
JC_1208	same ID			PCI-009	PCI-009				same ID	
JC_11034										
JC_11066										
JC_10198				UFC-003	UFC-003				same ID	
JC_10213				CEI-004	CEI-004					
JC_10215				CEI-005	CEI-005					
JC_10217				PCI-012	PCI-012					
JC_10249				UFC-009	UFC-009					
JC_10258				PCI-024	PCI-024					

JC_10334		CEI-011	CEI-011				
JC_10617							
JC_11296							
JC_11579							
JC_11623							
KS_11531							
JC_10151		PCI-006	PCI-006			same ID	
JC_10191		PCI-017	PCI-017			same ID	
JC_10248		CEI-008	CEI-008			same ID	
JC_10310		CEI-002	CEI-002			same ID	
JC_10416		CEI-009	CEI-009				
JC_10424		PCI-020	PCI-020				
JC_11062						same ID*	
KS_10337				same ID			same ID
JC_10142		PCI-011	PCI-011				
JC_10195		UFC-006	UFC-006			same ID	
JC_10214		PCI-019					
JC_10300		BPE-018	BPE-018				
JC_10968		PCI-034					
JC_10999						same ID*	
JC_11060							
JC_11401							
JC_10024		PCI-001	PCI-001			same ID	
JC_10089	same ID	PCI-008	PCI-008		same ID	same ID	
JC_10025		PCI-002	PCI-002				
JC_10168		CEI-003	CEI-003				
JC_10250		UFC-008	UFC-008				
JC_10257		UFC-005	UFC-005				
JC_10490							
JC_10551				same ID			
JC_11036							
KS_11453							

JC_10256	UFC-007	UFC-007
JC_10029	PCI-005	PCI-005
JC_10953	CEI-016	
JC_11000		
JC_11061		
JC_11297		

*Unpublished at time of analysis

References

1. Dubis AM, Cooper RF, Aboshiha J, et al. Genotype-dependent variability in residual cone structure in achromatopsia: towards developing metrics for assessing cone health. *Invest Ophthalmol Vis Sci* 2014;55:7303-7311.
2. Scoles D, Sulai YN, Langlo CS, et al. In vivo imaging of human cone photoreceptor inner segments. *Invest Ophthalmol Vis Sci* 2014;55:4244-4251.
3. Abozaid MA, Langlo CS, Dubis AM, Michaelides M, Tarima S, Carroll J. Reliability and repeatability of cone density measurements in patients with congenital achromatopsia. *Adv Exp Med Biol* 2016;854:277-283.
4. Langlo CS, Patterson EJ, Higgins BP, et al. Residual foveal cone structure in *CNGB3*-associated achromatopsia. *Invest Ophthalmol Vis Sci* 2016;57:3984-3995.
5. Langlo CS, Erker LR, Parker M, et al. Repeatability and longitudinal assessment of foveal cone structure in *CNGB3*-associated achromatopsia. *Retina* 2017;37:1956-1966.
6. Georgiou M, Litts KM, Kalitzeos A, et al. Adaptive optics retinal imaging in *CNGA3*-associated achromatopsia: Retinal characterization, interocular symmetry, and intrafamilial variability. *Invest Ophthalmol Vis Sci* 2019;60:383-396.
7. Mastey RR, Gaffney M, Litts KM, et al. Assessing the interocular symmetry of foveal outer nuclear layer thickness in achromatopsia. *Transl Vis Sci Technol* 2019;8:21.
8. Mastey RR, Georgiou M, Langlo CS, et al. Characterization of retinal structure in *ATF6*-associated achromatopsia. *Invest Ophthalmol Vis Sci* 2019;60:2631-2640.
9. Litts KM, Georgiou M, Langlo CS, et al. Interocular symmetry of foveal cone topography in congenital achromatopsia. *Curr Eye Res* 2020;45:1257-1264.
10. Georgiou M, Litts KM, Singh N, et al. Intraobserver repeatability and interobserver reproducibility of foveal cone density measurements in CNGA3- and CNGB3-achromatopsia. *Transl Vis Sci Technol* 2020;9:37.
